# Exploring the socio-ecological factors behind the (in)active lifestyles of Spanish post-bariatric surgery patients

**DOI:** 10.1080/17482631.2019.1626180

**Published:** 2019-06-12

**Authors:** Vicente J. Beltrán-Carrillo, Alejandro Jiménez-Loaisa, George Jennings, David González-Cutre, Natalia Navarro-Espejo, Eduardo Cervelló

**Affiliations:** a Department of Sport Sciences, Sport Research Centre, Miguel Hernández University, Elche, Spain; b Cardiff School of Sport and Health Sciences, Cardiff Metropolitan University, Cardiff, UK; c Department of Mental Health, Hospital Universitario del Vinalopó, Elche, Spain

**Keywords:** Physical activity, exercise, obesity, environment, barriers, facilitators

## Abstract

**Purpose**: Physical activity (PA) is considered essential for the treatment of morbid obesity and the optimization of bariatric surgery outcomes. The objective of this article was to identify the facilitators and barriers that bariatric patients perceived to do PA one year after finishing a PA programme for the promotion of a long-term active lifestyle. This objective was addressed from a socio-ecological and qualitative perspective.

**Methods**: Nine patients (eight women and one man), aged between 31 and 59 years, participated in semi-structured interviews directly following the PA programme and one year after it. A content analysis was carried out to analyze the qualitative data.

**Results**: Weight loss, improvement of physical fitness, perceived competence, and enjoyment were the main facilitators of PA. Complexes related to skin folds, osteoarthritis, perceived unfavourable weather conditions, lack of social support and economic resources, long workdays, lack of specific PA programmes, and other passive leisure preferences were the main barriers to participate in PA.

**Conclusions**: Results highlight the important interplay between personal, social environmental, and physical environmental factors to explain (in)active behaviours of bariatric patients. The findings of this article could be useful for future research and interventions aimed at promoting PA in bariatric patients.

## 1. Introduction

After the inefficacy of non-invasive methods for the treatment of morbid obesity, bariatric surgery has been shown to be an effective treatment option for long-term weight loss, improving medical comorbidities and psychological and social functioning after surgery (White et al., 2015). However, research has suggested that 10–25% of bariatric patients tend to have weight regain, and 5–25% may have complications postoperatively because of poor adherence to treatment recommendations (Sarwer, Dilks, & West-Smith, 2011). For that reason, successful bariatric surgery requires significant lifestyle and behavioural changes that include an appropriate diet and sufficient participation in physical activity (PA) (Bagdade & Grothe, 2012).

The role of PA in optimizing bariatric surgery outcomes has been receiving growing attention, and it is considered an important adjunct in the treatment of severe obesity (Herman, Carver, Christou, & Andersen, 2014). PA has been found to be a determinant of the long-term maintenance of weight loss after surgery (Moya et al., 2014), avoiding weight regain and the return of comorbidities associated with this condition (Sjöström et al., 2004). Moreover, it is also an important contributor to the prevention or treatment of psychological disorders such as depression or anxiety, and it is associated with improved quality of life after surgery (Jiménez-Loaisa, Beltrán-Carrillo, González-Cutre, & Cervelló, 2015).

The review article by Rudolph and Hilbert (2013) showed that numerous intervention studies have attempted to promote the behaviour of PA in bariatric patients. These studies included individual or group education sessions between patients and psychologists and/or dietitians periodically over time, in order to provide them with information for lifestyle changes. Bariatric patients who received a post-surgery behavioural lifestyle intervention increased their PA levels, in comparison with patients who did not receive an intervention (Rudolph & Hilbert, 2013).

Kalarchian and Marcus (2015), in another review of intervention studies with bariatric patients, pointed out that some studies included the patients’ participation in PA programmes. However, those programmes were mainly aimed at improving physical parameters, and did not include a psychological intervention to maintain an active lifestyle after the PA programme (Kalarchian & Marcus, 2015). For this reason, our research project included a PA programme based on motivational strategies for the promotion of a sustained active lifestyle (see Methods). These motivational strategies derived from self-determination theory (SDT) (Deci & Ryan, 2008). SDT is a theory of human motivation which assumes the existence of three basic psychological needs: autonomy, competence, and relatedness. Autonomy refers to the need to have control over our own behaviour and to make our own decisions. Competence describes the need to feel effective and successful in our interactions with the environment. Relatedness refers to the need to establish good social relationships, and to feel respected and valued by others. According to SDT, if basic psychological needs are satisfied in social contexts, people tend to be more autonomously motivated for the maintenance of healthy behaviours and their psychological wellbeing tends to improve (Ryan & Deci, 2017). A previous study derived from our research project showed that a PA program based in SDT can promote, in bariatric patients, enjoyment, happiness, self-confidence, motivation and intention to be physically active in the future (González-Cutre, Megías, Beltrán-Carrillo, Cervelló, & Spray, 2018).

However, previous research has also shown that bariatric patients rarely perform the PA they intended (Bond et al., 2013). The long-term PA adherence is a difficult matter, and the studies which analyze the facilitators and barriers to PA in bariatric patients are necessary to improve the interventions for the promotion of active lifestyles in this population (Dikareva, Harvey, Cicchillitti, Bartlett, & Andersen, 2016).

In recent years, few studies have shed light on the factors influencing PA participation in bariatric patients. For instance, Bergh, Kvalem, Mala, Hansen, and Sniehotta (2017) showed that being single, higher education level, and greater self-regulation predicted PA. Reid et al. (2016) indicated that neighbourhood walkability did not influence either daily steps or sedentary time. From a qualitative perspective, Wiklund, Fagevik, Olbers, and Willén (2014) pointed out that some physical side effects of bariatric surgery, such as diarrhoea or excess skin, prevented patients from being as active as they would like to be. On the contrary, weight loss, increased vitality, and improved ability after surgery favoured PA participation. The support from friends, family and health professionals was also perceived as necessary to be physically active. Another qualitative study (Dikareva et al., 2016), showed that feelings of embarrassment, and discomfort about their bodies are frequent among bariatric patients, especially when they feel socially exposed. Other perceived barriers were physical pain and lack of physical fitness. Some facilitators to PA were social support and access to exercise knowledge.

In spite of this previous evidence, this topic still needs further research (Dikareva et al., 2016), and more quantitative and qualitative studies are necessary. Qualitative studies can be of particular interest to understand the thoughts, feelings and behaviours of a small number of individuals in relation to social processes and particular contexts (Sparkes & Smith, 2014). Moreover, whereas quantitative studies measure variables which are preconceived by researchers, qualitative studies favour the emergence of unknown or unexpected information, giving voice to participants who can report their thoughts and experiences after open questions (Sparkes & Smith, 2014). Thus, in this emergent phase of the research line, qualitative studies could be useful for the identification of factors influencing PA in bariatric patients (Dikareva et al., 2016). This information could serve as fundament for future quantitative studies, in order to calculate the weight of the factors influencing PA in large samples of bariatric patients.

A socio-ecological approach has been considered an appropriate model to analyze the factors related to PA in different populations, such as children (Hesketh et al., 2017), adolescents (Devís-Devís, Beltrán-Carrillo, & Peiró-Velert, 2015), or women with overweight and obesity (Mama et al., 2015). This approach highlights the influence of personal (e.g., weight, perceived competence), social environmental (e.g., support from friends and family), and physical environmental factors (e.g., availability of PA facilities and natural spaces, weather conditions) on PA participation. To our knowledge, only one previous study has used a socio-ecological perspective to analyze the factors influencing PA in bariatric patients (Dikareva et al., 2016). The study (Dikareva et al., 2016) employed a qualitative methodology and carried out research with bariatric patients recruited from a clinic by poster advertising and word of mouth recommendations, but the participants were not previously involved in any intervention for the promotion of PA.

The aim of this study was to analyze, from a qualitative and socio-ecological perspective, the factors influencing that bariatric patients continue doing (or give up) PA one year after finishing a PA programme. This PA programme included a motivational intervention for the promotion of a sustained active lifestyle (see Methods).

## 2. Methods

### 2.1. Participants

The participants in this qualitative study were nine patients aged between 31 and 59 years (*M* = 46.77, *SD* = 9.75), who attended a PA programme one month after receiving bariatric surgery. They were mostly women (see Table I), as it is normally the case in Spain and other countries (Fuchs et al., 2015; Lecube et al., 2016). The participants were recruited from the same hospital by their clinical psychologist. The inclusion criteria for bariatric surgery included having a body mass index (BMI) greater than 40 kg/m^2^, or greater than 35 kg/m^2^ with associated co‐morbidity, and having no medical, psychological or social contraindication for surgery. The participation in the PA program after surgery was optional, and all participants accepted to take part in it before surgery. The PA programme was free for the participants and funded with the resources of the study (see section Funding).

**Table I.** Characteristics of participants.

Pseudonym	Gender	Age	Occupation	Comorbidities (pre-surgery)	BMI (pre-surgery)	BMI (1-month post-surgery)	BMI (post-PAP)	BMI (1-year post-PAP)
Telma	Female	31	Cobbler	No comorbidities	45.3	41.3	37.1	35.5
Alice	Female	31	Hairdresser	No comorbidities	38.2	34.3	25.9	26.9
Pam	Female	45	Cobbler	HTA	40.7	36.9	27.1	27.4
Emily	Female	49	Homemaker	HTA	38.4	35.6	29.1	29.0
Susan	Female	50	Cleaner	DL	40.8	37.6	27.1	24.8
Lucy	Female	53	Cobbler	HTA, DL	44.1	41.3	25.8	23.4
Lezly	Female	54	Psychiatrist	HC	43.1	39.8	33.5	31.6
Sofie	Female	59	Homemaker	No comorbidities	38.7	34.7	30.9	31.1
Andrew	Male	49	Taxi driver	HTA	45.1	38.7	27.2	24.5

BMI, Body Mass Index; PAP, Physical Activity Programme; HTA, Hypertension; DL, Dyslipidemia; HC, Hypercholesterolemia.

As the study was focused on information which was personal and private or contained valuations of other people, patients’ anonymity was preserved using pseudonyms. This study was carried out in Elche, a city located in the province of Alicante (Valencian Community, Spain). The economy of this city is mainly dependant on the footwear industry and tourism. The majority of population pertain to a middle socioeconomic status.

### 2.2. Procedure and interview protocol

The PA programme took place in a public fitness centre located in Elche and lasted six months. The instructors were exercise and sport sciences professionals (degree in sport sciences, master’s degree in physical activity and health), who were trained in strategies based on SDT (Deci & Ryan, 2008) focused on the satisfaction of needs for competence, autonomy and relatedness during the sessions (see Table II). The programme consisted of two sessions per week during the first two months, three sessions per week during the intermediate two months, and four sessions per week during the last two months, each lasting 1 hour and 30 minutes. More information about the PA programme can be found in González-Cutre et al. (2018).

**Table II.** Examples of strategies based on SDT applied by instructors.

Need	Strategies
Autonomy	To give options to choose different activities, machines and working ranges. Patients received training on how to do healthy exercise by themselves. To ask participants for their opinion on the activities.
Competence	To provide positive feedback and information to the patients about their progress. To establish short-term goals so that patients assess their progress every month. To follow-up an individualized programme with achievable objectives.
Relatedness	To propose physical and nonphysical tasks in which patients had to interact. Participants were encouraged to correct execution of exercises among them. The instructors smiled, supported, and encouraged patients. A caring climate was created, in which the instructors showed interested about the patients’ lives.

The research project was approved by the Ethical Research Board of the first author’s university (Code: DPS-DGC-001–11; Date of approval: 2 February 2012). The PA programme was evaluated and approved by a medical council formed by doctors, surgeons, endocrinologists, nutritionists, psychologists, psychiatrists and exercise science professionals. Participants were informed about the aims and procedure of the study and provided written consent. In-depth semi-structured interviews were conducted and recorded with each participant to gather qualitative information. The interviews took place in a quiet room at the first author’s research centre. Firstly, semi-structured interviews were conducted just after the PA programme (July 2012). These interviews were conducted, regarding the purpose of this article, to know if the participants had the intention of participating in PA in the future, after the end of the PA programme. Secondly, semi-structured interviews were conducted again one year after the end of the PA programme (July 2013) with the same patients with the dual purpose of (1) knowing if participants had performed PA during that year and (2) identifying the factors that favoured or hindered their participation in PA during the year. The interviews lasted between 40 and 60 minutes. An example of the initial questions guiding the first and second interviews is shown in Table III.

**Table III.** Example of the initial questions guiding the interviews.

Interview	Questions
First interview (just after the PA programme)	Do you think the PA programme will cause changes in your PA habits in the future?
	During the PA programme, did you receive enough information and knowledge to do exercise and keep an active lifestyle after finishing the PA programme?
	Are you going to continue doing PA after the PA programme?
Second interview (one year after the end of the PA programme)	Have you performed physical activity after the PA programme? Why?
	Why do you engage with PA now?
	After the PA programme, what barriers have hindered your participation in PA? What difficulties have you found to do PA?
	What do you think you would need to continue practicing PA?
	Would you return to the PA programme if this was done again?
	Do you think these types of PA programmes should be funded by the public health system or by the participants? Why?

PA, Physical Activity.

### 2.3. Data analysis

Semi-structured interviews were transcribed by the interviewer with a word processor software immediately after conducting them. Transcriptions were analyzed with the support of the software NVivo, which was used to organize and classify data efficiently (Bazeley & Jackson, 2013).

The qualitative data were analyzed following combined strategies of both “conventional” (inductive) and “directed” (deductive) content analysis (Hsieh & Shannon, 2005). The analysis started with an inductive phase to ensure that any information that could shed light on the purpose of the study was included in the analysis. Concretely, all transcriptions were read several times to become familiar with the data and achieve a sense of the whole. Second, those text fragments that captured key thoughts or concepts related to the factors influencing that bariatric patients were active or inactive were identified and coded. Third, these codes (the text fragments labelled with the thoughts they illustrated) were classified, using inductive reasoning, into a map of interrelated categories and subcategories which emerged when codes were compared to identify similarities, differences and relationships. Then, in a deductive phase of the analysis, the research team checked that a socio-ecological approach fitted well with the data, and was useful to interpret, report and discuss the data without discarding any important information. In fact, a socio-ecological approach was finally considered a more appropriate theoretical framework to analyze the data than SDT, although SDT guided the motivational strategies included in the PA programme.

The process of data analysis, led by the second author of this article, was supervised by the other members of the research group, who played the role of “critical friends” (Smith & McGannon, 2018). The members of this research group were different from the instructors of the PA programme. This group consisted of a clinical psychologist, sport sciences researchers, and experts in qualitative methods. During a series of three meetings, the leader of the analysis presented the data analysis using diagrams, outlined the codes included in the different categories, and responded to the questions and suggestions of the critical friends. The critical friends help their colleague to refine the names and contents of the different categories and to arrive to a more coherent system of categories. The final system of categories sustained the headlines and structure of results presented in the following section. The critical friends also collaborated to improve the write-up of the analysis and the entire article. The involvement of critical friends during the process of data analysis encouraged the quality of interpretations and favoured a more rigorous and plausible data analysis (Smith & McGannon, 2018).

## 3. Results and discussion

Three main categories derived from our content analysis: 1) Bariatric patients’ intention of participating in PA just after the PA programme; 2) Facilitators to PA one year after the end of the PA programme; and 3) Barriers to PA one year after the end of the PA programme. These categories, with their corresponding subcategories, are described and discussed in the following sections.

### 3.1. Bariatric patients’ intention of participating in PA just after the PA programme

Just after the PA programme, all bariatric patients who participated in this study reported their intention of engaging in PA in the future:

Telma: I’m sure that exercise will be part of my life. In fact, I have already bought a bike for me. And I have started to walk and even run, something I had never done in my life. Because I don’t want to get fat again. I’ve not doubt about it. Would I go back to the way I was? No way!

It is also noteworthy that all participants argued that the programme had given them sufficient knowledge to do PA without having large resources or without joining a gym, because of all the talks, advice and practical sessions that the instructors provided them:

Pam: They [instructors] have given us all the options. Whoever doesn’t want to do physical activity now, it’s because they don’t want to. You cannot say: “Oh, I don’t have money to go to the gym!” Well, you don’t have money to go to the gym, but you can do it on the street.

From a socio-ecological perspective, the intention to be active and having knowledge to be active are two personal factors related to PA participation, as other studies have pointed out (Dikareva et al., 2016; Peacock, Sloan, & Cripps, 2014; Wiklund et al., 2014). The role of the PA programme instructors, who provided support and knowledge to be active, can be considered a potent socio-environmental factor for the promotion of an active lifestyle.

### 3.2. Facilitators to PA one year after the end of the PA programme

#### 3.2.1. The perceived benefits of PA

In line with previous research with bariatric patients (Wiklund et al., 2014), weight loss and maintenance and improvements in health and physical function were the main reasons that led participants to do PA:

Lezly: Because I know it’s necessary … I have been able to lose and control my weight doing exercise … so I think it’s very important.

Pam: [I do PA] … for my health. To feel better, agile, not so exhausted, to climb stairs that I couldn’t climb before … It was at least ten years ago since I last came from my sister’s house because she lives on the fourth floor, and she has no lift.

Weight loss and the improvement of physical fitness increased participants’ perceived competence. From a socio-ecological approach, perceived physical competence represents a personal factor influencing PA participation, as a previous study with bariatric patients has shown (Wiklund et al., 2014). A higher physical competence also permitted participants to interact more successfully with their physical environment (e.g., being able to go up to the fourth floor of an apartment block), improving at the same time their interactions with their social environment (e.g., being able to visit a relative). Moreover, improved physical competence allowed participants to enjoy PA:

Andrew: What used to be hard for me, it isn’t now. Before [the surgery and PA programme] I walked ten minutes and I was exhausted. Now, ten minutes are like going down the stairs of my house. Or running ten minutes … before I was even wanting to throw up, and now for me it’s very little effort. What I am doing now was unthinkable before. Now, I have fun, I have a good time [doing PA].

Previous research has shown that lack of enjoyment is a common obstacle for bariatric patients to do PA (Peacock et al., 2014), whereas perceived physical competence promotes a high intrinsic motivation to PA (doing PA because you like and enjoy it) and adherence to exercise (Silva et al., 2008).

It is also remarkable how, through weight loss, some patients improved their physical self-concept and self-esteem, thus reducing their previous insecurity and avoidance of social relationships:

Lucy: [Now] I feel comfortable. Before … I avoided people. I always had excuses … “my legs hurt, my head hurts, I’m not well … ” Now I like to get involved. I mean, right now, I’m always thinking about making trips or going out on the weekends with my friends … they call me, or I call them … I avoided this kind of activities before. I don’t know if I was ashamed of myself.

This kind of psychological (personal) benefits can also favour a better interaction with the social environment and a higher involvement in social activities which can involve PA.

#### 3.2.2. Social support

From a socio-ecological perspective, social support is considered an important socio-environmental factor that fosters PA (Giles-Corti & Donovan, 2002). In this regard, the participants of this study declared that the support of family and friends was crucial to be active. For instance, Susan said that her son helped her to do PA at home every day, because “they were fond of playing in the pool”. Andrew reported cycling because he liked “going out with friends and knowing new places”. These findings are in line with other studies with bariatric patients which have previously highlighted the importance of social support to acquire an active lifestyle (King & Bond, 2013). In Andrew’s case, as knowing new places was also a stimulus for him to do cycling, a mixed influence of social and physical environmental factors on his PA behaviour can be observed.

### 3.3. Barriers to PA one year after the end of the PA programme

#### 3.3.1. Skin folds and body complexes

Massive weight loss following bariatric surgery frequently results in large skin folds. The excess of skin usually has a negative impact on bariatric patients’ quality of life, self-esteem, body image, and physical functioning (Klassen, Cano, Scott, Johnson, & Pusic, 2012). Among our participants, skin folds represented a source of new body complexes that did not exist pre-surgery:

Telma: Now I’ve more [body] complexes. Before, I went to the beach in bikini and I even went topless … And now if I’m going to the beach … if I could go with a burka, I would go with a burka. I swear, now I have more complexes, look [the interviewee shows her skin folds].

Other studies have also found that body complexes with skin folds are common among bariatric patients, and that these body complexes are perceived as a barrier to PA when bariatric patients feel socially exposed in activities like swimming (Biörserud, Olbers, & Fagevik-Olsén, 2011; Wiklund et al., 2014).

#### 3.3.2. Knee and hip osteoarthritis

Joint pain is an extensive problem for bariatric patients, due to severe obesity prior to surgery. Although knee and hip pain as a cause of osteoarthritis decreases after bariatric surgery (King et al., 2016), these problems frequently remain (Vincent et al., 2012). In our study, two women reported pain in their knees and one woman in her hip, and this was a personal factor leading to stop walking during specific periods of time, which could be specific days or even full weeks sometimes:

Susan: I stopped during a period … because my knee hurt a lot, and I took a one-week rest.

#### 3.3.3. Coping with unfavourable weather conditions

Some of our participants reported that unfavourable weather conditions in the province of Alicante, such as fluctuations in the heat, cold or wind, were barriers to their participation in PA:

Emily: If it’s raining, I don’t go for a walk, if it’s windy, I don’t go for a walk, if it’s hot, I don’t go for a walk. That’s why I say that, from January to now [July], I’ve walked in two months.

Difficulties with coping with the weather have been reported by bariatric patients in previous studies, but these barriers to PA seem to be more related to a “mental obstacle” than to important physical environmental barriers (Dikareva et al., 2016; Wiklund et al., 2014). It is important to note that all the participants in our study lived in the province of Alicante, a Spanish Mediterranean area characterized by having a mild weather throughout the year (information available in *www.tutiempo.net*). Although summers are hot, the weather does not seem to be a real barrier to do PA for the most part of the year.

#### 3.3.4. Lack of social support

Some participants reported lack of social support from family or friends to be active. They missed having someone to encourage and to coerce them to do PA as when they were involved in the PA programme:

Emily: I would like my daughter or friends to come and tell me “come on, let’s go!” I don’t have it … I would like them to encourage me to start [to walk]. Many times, I don’t have anyone to commit to or anyone who comes to pick me up … so when I have a minimal excuse, I don’t go for a walk.

Social support is essential to acquire an active lifestyle in these patients (King & Bond, 2013). Support from friends and family and having a companion for PA have consistently emerged as important correlates of leisure-time PA and walking in adults (Giles-Corti & Donovan, 2002; Shelton et al., 2011). Nevertheless, it is usual that family members of bariatric patients also cope with obesity and related comorbidities and demonstrate high levels of sedentary behaviour (Lent et al., 2016).

#### 3.3.5. Lack of economic resources

The lack of economic resources was perceived by most participants as a barrier to engage in PA. They reported being in a delicate economic situation because they and/or their spouses were unemployed and, as a result, it was impossible to join a gym. They therefore had to opt for inexpensive activities such as walking:

Emily: I go for a walk, because in our city the gyms are very expensive … my husband is unemployed, and I don’t work.

Susan: I have walked [after the PA programme]. Unfortunately, the problem is the money … I cannot afford the gym. My husband doesn’t work for most of the year, so we cannot spend that money because we need it to pay the house.

Financial reasons have emerged in other studies with bariatric patients as an obstacle that prevent or difficult participation in PA (Wiklund et al., 2014). Moreover, unemployment has been associated with increased physical inactivity, especially in women (Macassa et al., 2016). Some of the participants in this study worked in the footwear sector, which was severely punished during the economic crisis in Spain (about 50% of reduction in the size of this sector and 24% of total loss of employment) (Albertos-Puebla & Sánchez-Hernández, 2014). This was the case of Emily, who worked in this sector before losing her job.

#### 3.3.6. Long workdays

“Lack of time” and “being exhausted” due to long workdays also emerged as important perceived difficulties to be active:

Telma: I get up at five in the morning, to be at six at work, and I finish at nine p.m. and when I arrive at home, it’s ten p.m. When do I take the bike? On Saturdays, I go to work at six in the morning and I finish at four p.m. … While I arrive at home it’s five p.m. Exhausted after all week, I don’t feel like cycling.

A previous study also showed that having a job is sometimes perceived by bariatric patients as an obstacle to engage in PA, especially in women (Durand-Moreau et al., 2015). In this regard, our study was developed in a context of economic crisis, where women of Southern European countries (such as Spain) had to work long hours to reduce their family’s financial stress and ordinarily tended to perform more domestic workload than men (Artazcoz et al., 2016). These social environment factors could increase women’s perceived personal barriers to be active, as lack of time or lack of energy/vitality during the scarce leisure time.

Nevertheless, the influence of other personal factors should be considered when some participants in this study understood having a job as an obstacle to do PA. It is possible that “lack of time” hid other perceived personal obstacles to be active, such as boredom, lack of priority or lack of enjoyment with PA participation, as other studies with bariatric patients have found (Peacock et al., 2014; Wiklund et al., 2014). The fatigue associated with an obese condition could be another personal factor influencing that long working hours became a barrier to PA participation (Resnick, Carter, Aloia, & Phillips, 2006). Moreover, the ending of the PA programme, mentioned in next section, could represent another socio-environmental factor hindering PA participation. The participants had attended a PA programme several times a week. One year later, some of them reported lack of time as a barrier to PA. Perhaps, PA participation with perceived lack of time is a matter of priority, and PA options need to be attractive enough to encourage participation.

#### 3.3.7. Lack of specific PA programmes for this population

To the question “Would you return to the PA programme if this was done again?” all patients responded affirmatively (e.g., “Of course”, “yes, with my eyes closed”). Unfortunately, after a deliberate six months of intervention, the programme of the study ended, and the participants expressed their concern:

Sofie: This is a disease and the government should pay [the costs of the PA programme] … We have been operated and the programme has benefited us for four or five months, then we should keep on … it’s like when we get a candy and then they remove it. If I have become very good in five months … and I cannot pay, I cannot do more exercise, I’m back again. Well, what do we do?

The participants of our study thought that attending sport and PA facilities was expensive, in line with previous research with bariatric patients (Wiklund et al., 2014). With regard to this economic barrier, the study carried out by Tumiati et al. (2008) showed that cheaper options for PA, such as home-based individualised physical fitness programmes, can be motivating for obese patients to do PA. Nevertheless, in the context in which this study was developed, the public health system did not include specific PA programmes or counselling PA services for this population (Jiménez-Loaisa et al., 2015). The programme of the study ended, and some patients felt abandoned. The lack of resources, staff and facilities to offer long-term PA programmes for these patients was a social and physical environmental factor hindering their PA participation.

#### 3.3.8. Other passive leisure preferences

Sometimes, PA was not the favourite leisure option for bariatric patients. Some participants stated a preference for other leisure activities, especially in summer and weekends:

Telma: And on Sundays, now that it’s summer, have I to ride a bike? I prefer to go to the beach. Honestly, it’s true.

A study carried out with bariatric surgery candidates (Zabatiero et al., 2016) showed that participants also preferred to engage in sedentary leisure activities, such as watching TV or sleeping, rather than taking part in programmed exercise during their leisure time.

## 4. Conclusions and recommendations

The findings of this study revealed that both individual (in a psycho-physical sense) and environment (understood as a blend of familial, social, economic and atmospheric conditions) played a major role in the consolidation of (in)active lifestyles. A qualitative socio-ecological perspective was useful to identify the interrelated influence of personal, social environmental and physical environmental factors on PA participation.

The qualitative design and small sample of our study were not aimed at generalizing results. However, our qualitative design let the collection of in depth information about the participants, and content analysis made possible the emergence of insights which can be transferable and useful for future research and interventions aimed at promoting PA in bariatric patients.

After the PA programme, all patients intended to be physically active in the future and argued to have enough knowledge to do PA on their own. Nevertheless, one year later, our results pointed out that the move from an intention to the actual behaviour of PA is a difficult step to take.

Weight loss and maintenance, coupled with perceived improvements in physical fitness, gave rise to a greater perception of physical competence, greater enjoyment with PA and improvements in the interaction with the physical and social environment. All these factors, together with social support, were identified as facilitators to PA one year after the PA programme.

Nevertheless, body complexes caused by skin folds can be a personal barrier to PA in activities requiring revealing clothes, in which participants can feel socially exposed (e.g., activities in the aquatic environments, stroll along the beach, etc.). In this regard, it is of utmost importance that the closest social environment of these patients (health professionals, family, friends, etc.) emphasizes the importance of health and quality of life over aesthetics, to favour that patients are more satisfied with their body shape. Moreover, it would be desirable to guarantee, in PA contexts, inclusive social environments in which exercisers are respectful of other people’s appearances. The physical environment should also be inclusive. Exercise facilities should possess specific designs and physical structures to preserve patients’ privacy if they have some body complex and do not want to feel observed by others (e.g., individual showers, swimming pools or fitness rooms closed to external observers).

The pain associated with knee or hip osteoarthritis was another barrier to PA. Perhaps, instead of avoiding PA, bariatric patients need to know what type of exercise they should do to avoid pain, favour rehabilitation, and improve their physical functioning. The exercise counselling by qualified professionals seems necessary for bariatric patients. Coping with unfavourable weather conditions was also reported as a barrier to PA. However, the climate of Alicante (Spain) does not seem to represent an important physical environmental barrier to PA. Lack of motivation, or other passive leisure preferences, may be also influencing bariatric patients’ perception of this barrier. Another perceived barrier to be active was lack of social support. Future interventions for the promotion of PA might be also focused on patients’ relatives and friends, as a way to enhance more active and supporting social environments.

The lack of economic resources and unemployment were perceived by bariatric patients as obstacles to do PA. Health education focused on healthy habits and PA promotion would be necessary so that unemployed bariatric patients find economical ways to be active. At the same time, some participants perceived the lack of time and exhaustion associated with long workdays as a difficulty to be active. In order to promote PA, employers should make efforts to avoid very long working hours and work-overload for their workers, which seems quite incompatible with an active lifestyle. Companies could also promote active recess especially in those employees who work sitting (e.g., the footwear industry), enable space for exercise in the company facilities or near to them, or contracting a sport sciences professional who could supervise personal or group-based exercise programmes for company employees.

The end of the PA programme represented one of the most important barriers for the patients to continue with an active lifestyle. It would be beneficial for public health systems to include PA programmes and advisory exercise services for these patients. This action requires building bridges of relationships between medical staff and exercise and sport sciences professionals. Moreover, it would be necessary to investigate the cost-benefit of implementing these services and explore ways to make them economically sustainable. In this regard, previous research has pointed out that the investment in the promotion of PA is necessary to reduce the economic costs of the diseases related to physical inactivity in the health systems (Kruk, 2014).
